# Highly Efficient Differentiation and Enrichment of Spinal Motor Neurons Derived from Human and Monkey Embryonic Stem Cells

**DOI:** 10.1371/journal.pone.0006722

**Published:** 2009-08-24

**Authors:** Tamaki Wada, Makoto Honda, Itsunari Minami, Norie Tooi, Yuji Amagai, Norio Nakatsuji, Kazuhiro Aiba

**Affiliations:** 1 Stem Cell and Drug Discovery Institute (SCDI), Shimogyo-ku, Kyoto, Japan; 2 Institute for Integrated Cell-Material Sciences (iCeMS), Kyoto University, Sakyo-ku, Kyoto, Japan; 3 Institute for Frontier Medical Sciences, Kyoto University, Sakyo-ku, Kyoto, Japan; City of Hope Medical Center, United States of America

## Abstract

**Background:**

There are no cures or efficacious treatments for severe motor neuron diseases. It is extremely difficult to obtain naïve spinal motor neurons (sMNs) from human tissues for research due to both technical and ethical reasons. Human embryonic stem cells (hESCs) are alternative sources. Several methods for MN differentiation have been reported. However, efficient production of naïve sMNs and culture cost were not taken into consideration in most of the methods.

**Methods/Principal Findings:**

We aimed to establish protocols for efficient production and enrichment of sMNs derived from pluripotent stem cells. Nestin+ neural stem cell (NSC) clusters were induced by Noggin or a small molecule inhibitor of BMP signaling. After dissociation of NSC clusters, neurospheres were formed in a floating culture containing FGF2. The number of NSCs in neurospheres could be expanded more than 30-fold via several passages. More than 33% of HB9+ sMN progenitor cells were observed after differentiation of dissociated neurospheres by all-trans retinoic acid (ATRA) and a Shh agonist for another week on monolayer culture. HB9+ sMN progenitor cells were enriched by gradient centrifugation up to 80% purity. These HB9+ cells differentiated into electrophysiologically functional cells and formed synapses with myotubes during a few weeks after ATRA/SAG treatment.

**Conclusions and Significance:**

The series of procedures we established here, namely neural induction, NSC expansion, sMN differentiation and sMN purification, can provide large quantities of naïve sMNs derived from human and monkey pluripotent stem cells. Using small molecule reagents, reduction of culture cost could be achieved.

## Introduction

Spinal motor neurons (sMNs) are cells in the central nervous system (CNS), project their axons outside the CNS and control muscles directly or indirectly through neuromuscular junctions. It is well known that differentiation of sMNs is spatio-temporally controlled in mouse embryonic brain development [1]–[9]. A combination of all-trans retinoic acid (ATRA) and sonic hedgehog (Shh) would be essential for inducing sMNs from neural stem cells (NSCs) with proper timing. *In vitro* sMN differentiation from both mouse embryonic stem (ES) cells [10], [11] and human embryonic stem cells (hESCs) [12], [13] has been reported by several groups recently. Mouse ES cell/hESC-derived sMNs could also be a useful model to investigate the gene regulation pathways and proteomics in CNS neuronal development.

Motor neuron diseases (MNDs) such as amyotrophic lateral sclerosis (ALS) and spinal muscular atrophy (SMA) are progressive neurological disorders and show severe symptoms in many cases. Currently there are no cures or efficacious treatments for MNDs. Transplantation of hESC-derived sMNs is a therapeutic method of clinical application, however, there are many challenging safety issues to be overcome. In contrast, generation of MND models could be useful in basic research for regenerative medicine and pharmaceutical applications, because ESCs have advantageous properties such as a uniform genetic background and the ability to differentiate into all cell types in high quantity and good quality [14].

A large-scale and low-cost culture system is required for research toward drug discovery and development. Neurosphere culture is a typical method for enhancing the numbers of brain-derived NSCs [15] though neurospheres are considered to be a heterogeneous population of multipotent NSCs and neural progenitor cells [16], [17]. Several growth factors, such as fibroblast growth factor type 2 (FGF2) and epidermal growth factor (EGF), are essential for proliferation or maintenance of NSCs [18]–[20]. Although both mouse and human ESC-derived NSCs could form neurosphere-like cell aggregates [21], [22], it is unknown whether large-scale culture of neurospheres is feasible for expansion of ESC-derived NSCs and maintenance of their ability to differentiate.

Small molecular compounds could be useful for constructing stable and low-cost culture systems. Several compounds have been reported as antagonists or agonists of intracellular signaling molecules such as Shh [23], FGF2 [24], or Wnt proteins [25]. Although the specificity of these compounds has been debated, the running cost can be reduced if the compounds function appropriately in differentiation of ESCs.

Techniques for isolating cells of interest from a heterogeneous bulk population are in demand because purity of cell population is important in many biological assays. Unfortunately, appropriate specific surface markers have not been identified for sMN sorting. Although mouse [7], [26] and human [27] sMNs could be purified using enhanced green fluorescence protein driven by a sMN-specific promoter, the purified sMNs are not already naïve cells because of ectopic transgene expression. The gradient centrifugation method has been used to isolate sMNs from embryonic chicks [28] and embryonic mouse spinal cords [29], [30], but there has been no report on using such method for isolation of human fetus- or hESC-derived sMNs.

Therefore, in this study we examined the highly efficient production and enrichment of sMNs derived from hESCs. Human ESCs were induced to differentiate into NSCs by a bone morphogenetic protein (BMP) antagonist, Noggin, or a small molecular compound, Dorsomorphin, in a monolayer culture system. These hESC-derived NSCs had rostral characteristics by default. NSCs differentiated into sMNs in medium containing ATRA and Shh or SAG, an agonist of Shh. Efficient expansions of sMNs were also achieved by passagable neurosphere suspension culture in the presence of FGF2. Moreover, sMNs were highly purified by gradient centrifugation, though further optimization was required for much higher efficiency. Using a combination of the techniques mentioned above, we established highly efficient sMN production from hESCs. These techniques were also cost- and time-effective improvements compared with those of previous reports.

## Materials and Methods

### Spinal Motor Neuron Induction from Human Embryonic Stem Cells

**Figure 1:**
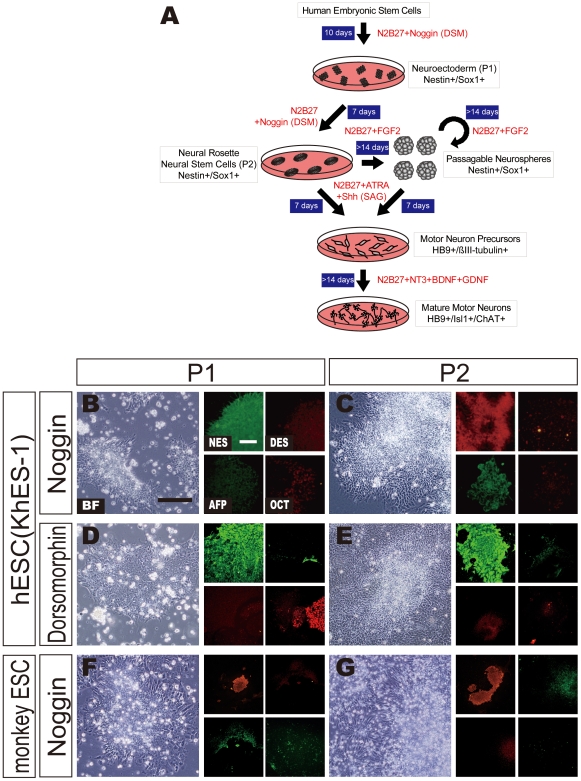
Noggin Treatment Induces Neural Differentiation of Primate Embryonic Stem Cells. (A) Schematic procedures of a highly efficient motor neuron differentiation system from primate embryonic stem cells are shown. Red text shows medium components and white text in blue boxes shows culture days. (B–G) KhES-1 human or CMK6 cynomolgus monkey ESCs were cultured in the presence of recombinant mouse Noggin for 10 days (Passage 1; P1, B and F) and 7 more days (Passage 2; P2, C and G). Cells were fixed with 4% PFA and stained with antibodies against specific markers: Nestin (NES: neuroectoderm), Desmin (DES: mesoderm), α-fetaprotein (AFP: endoderm), and Oct4 (OCT: undifferentiated ESC) at P1 and P2. Black bar in BF image and white bar in NES image indicate 25 µm (B). Dorsomorphin-induced neural differentiation is shown in D and E.

Human embryonic stem cells (hESCs; KhES-1, KhES-2, and KhES-3, passage numbers around 30–70) and cynomolgus monkey ESCs (CMK-6) were obtained from Institute for Frontier Medical Sciences, Kyoto University and Mitsubishi Tanabe Pharma Corporation, respectively. The hESCs were treated according to the guidelines for utilization of hESCs established by the Ministry of Education, Culture, Sports, Science and Technology, Japan. The procedure for maintenance of hESCs and monkey ESCs was essentially the same as described previously [31]. Briefly, hESCs or monkey ESCs were cultured on mitomycin C-treated mouse embryonic fibroblasts in primate ES medium (ReproCELL, Tokyo, Japan) supplemented with 5 ng/ml FGF2 (Wako, Japan). To obtain NSCs or neural progenitor cells derived from both ESCs, we modified the method of Gerrard *et al*. [32]. For neural induction, dissociated ES colonies 40–70 µm in diameter were selected with Cell Strainers (BD-Falcon, Franklin Lakes, NJ) and plated on poly-L-lysine/laminin (PLL/LM; both from Sigma-Aldrich, St. Louis, MO) -coated culture dishes in N2B27 neural differentiation medium (1∶1 mix of DMEM/F12 supplemented with N2 and Neurobasal medium supplemented with B27 [all from Gibco, Gland Island, NY], supplemented with 100 ng/ml mouse or human recombinant Noggin (R&D Systems) or 200 nM Dorsomorphin (BIOMOL, Plymouth Meeting, PA) as an inhibitor of BMP signaling for the first 10 days. The medium was changed every two days (P1 in Figure 1A). Primary colonies were split into small clumps by 200 U/ml Collagenase with 1 mM CaCl_2_, plated into new PLL/LM-coated culture dishes and cultured for another 7 days with N2B27 supplemented with BMP inhibitors (P2 in Figure 1A). Noggin-treated cells formed radial clusters, namely neural rosettes in the P2 stage (Figure 1).

For sMN differentiation, P2 cells were dissociated by Accutase (Innovative Cell Technologies, Inc., San Diego, CA) and cultured on PLL/LM plus human plasma Fibronectin (Chemicon, Billerica, MA) -coated culture dishes (PL/LM/FN) at 1000 cells/cm^2^ in N2B27 supplemented with ATRA (Sigma) and either human recombinant Shh (R&D Systems) or SAG (Alexis, San Diego, CA) for 7 days, and then cultured in N2B27 containing 10 ng/ml brain-derived neurotrophic factor (BDNF; R&D Systems), 10 ng/ml glial cell-derived neurotrophic factor (GDNF; R&D Systems) and 10 ng/ml neurotrophin-3 (NT-3; R&D Systems) for another 2 weeks or longer.

### Neurosphere Culture

NSCs were dissociated at the end of P2 and cultured at 10^6^ cells/ml with N2B27 in the presence of 10 ng/ml FGF2 and 50 ng/ml heparin (Sigma) for 2 weeks or longer. Neurospheres were collected by gentle centrifugation and incubated with Accutase for 10 minutes at 37°C. Cells were dissociated by 1 ml micropipette and washed with N2B27 medium to remove residual enzymes. Neurospheres were passaged several times in N2B27 with both FGF2 and epidermal growth factor (EGF; R&D Systems).

### Immunocytochemistry

Cells were fixed in freshly prepared 4% paraformaldehyde (PFA) for 10 minutes at room temperature (RT) and were permeabilized by 0.5% Triton-X (Wako Chemicals, Tokyo, Japan) for 5 minutes at RT. Primary antibodies used in this study included polyclonal antibodies against Nestin (1∶200; Chemicon), Sox1 (1∶200; Sigma), choline acetyltransferase (ChAT) (1∶100; Chemicon), Synapsin (1∶200; Chemicon), βIII-tubulin (1∶200; Abcam, Cambridge, MA), GFAP (1∶400; DAKO, Glostrup, Denmark), and Desmin (1∶200; Thermo Scientific, Waltham, MA) or monoclonal antibodies against Oct4 (1∶200; Santa Cruz, Santa Cruz, CA), βIII-tubulin (1∶200; Sigma), Nestin (1∶200; Chemicon), Olig2 (1∶200; IBL, Takasaki, Japan), MNR2 (HB9, 1∶5), Isl1 (1∶5; DSHB, Iowa City, IA), BF-1 (1∶400; Affinity BioReagents, Golden, CO), and AFP (1∶200; Sigma).

As secondary antibodies, goat anti-mouse immunoglobulin G Alexa Fluor 488 and 568, and goat anti-rabbit Alexa Fluor 488 and 568 (all at 1∶1000; Molecular Probes Inc., Eugene, OR) were used. Cells were counterstained with 4,6-diamidino-2-phenylindole (DAPI, Sigma) for visualization of all nuclei. Image acquisition was performed using an inverted Olympus IX71 epifluorescence microscope with appropriate filter sets (Olympus, Tokyo, Japan) using single-channel acquisition on an Olympus DP70 CCD camera with DP software version 2.1,1.83 (Olympus).

### Reverse Transcription-Polymerase Chain Reaction

Total RNA was extracted using RNeasy kits (Qiagen, Tokyo, Japan). Two micrograms of total RNA were used to synthesize cDNA in 20 µl reaction scales by High Capacity cDNA Reverse Transcription Kits (Applied Biosystems, Foster City, CA). An appropriate reaction volume (0.5 µl) was used for quantitative PCR (qtPCR) with SYBR Green Master Mix on a 7500 Real-time PCR System (Applied Biosystems). Relative gene expression levels were determined with the comparative C_T_ method in triplicate experiments. Primers used in this study are listed in Table S1.

### Purification of Motor Neurons by Gradient Centrifugation

To purify ESC-derived sMNs, we applied the procedure of Gingras *et al*. [29] with slight modification. sMNs were enzymatically dissociated by Accutase and washed with N2B27 once by centrifugation, and were resuspended in 3 ml of DMEM/F12 containing 0.004% DNaseI (Sigma Aldrich). The gradient reagent, Biocoll (Biochrom AG, Germany), was diluted to 1∶1.76 with L15 medium (Gibco) and 4 ml was put into 15 ml conical tubes (BD-Falcon). Cell suspension was gently placed over the gradient reagent. Schematic drawings of the overlays are shown in Figure S7A. After centrifugation of 500×g for 20 min at 4°C, cells at interfaces between gradients were collected and dissolved in N2B27 medium, and then cultured at an appropriate cell density with growth factors. After overnight culture, cells were fixed for measurement of sMN enrichment.

### ENStem-A Culture

H9 hESC-derived neural progenitor cells (ENStem-A™) were obtained from Millipore (Billerica, MA). ENStem-A cells were expanded according to the manufacturer's protocol. To form neurospheres, ENStem-A cells were cultivated in N2B27 medium containing 10 ng/ml FGF2 for 7 days at 10,000 cells/ml. Neurospheres were dissociated by Accutase and then plated on PLL/LM-coated dishes in N2B27 medium containing 10 µM ATRA and 10 nM SAG. The medium was changed every 3 days. After 2 weeks, cells were fixed for immunocytochemistry analysis.

### Patch-Clamp Recordings

Human ESC-derived sMNs, cultured for 14 days after ATRA/SAG treatment, were used for electrophysiological analysis. An HEKA EPC10 amplifier was used for recording of data in a whole-cell patch clamp configuration (HEKA Instruments Inc, Bellmore, NY). An external solution (140 mM NaCl, 5 mM KCl, 10 mM HEPES, 2 mM CaCl_2_, 1 mM MgCl_2_, and 10 mM glucose) was adjusted to pH 7.2 with NaOH. A pipette solution (147 mM CsCl, 5 mM EGTA, 2 mM ATP-Mg, and 10 mM HEPES) was adjusted to pH 7.2 with CsOH. Intracellular current injections were delivered via a recording pipette in whole-cell current-clamp recordings. The duration of the current pulse was 1 second, and the amplitude was 50 pA. In whole-cell voltage-clamp recordings of glutamate application experiments, sMNs were perfused with the external solution in the presence of 100 µM glutamate for 20 seconds. The current-voltage relationship was obtained by holding the membrane potential at −60 mV and then delivering a depolarizing pulse for 500 ms in a stepwise manner with 10-mV increments from −60 mV to 40 mV.

### Co-Culture Experiments with Skeletal Muscle Cells

Undifferentiated mouse myoblast C2C12 cells were maintained in DMEM with 20% FBS. Co-culture procedures were carried our according to the previous report [33]. C2C12 cells were differentiated in DMEM with 2% horse serum (Sigma) for 4 days. C2C12-derived myotubes were co-cultured with hESC-derived sMNs in N2B27 medium for 4 to 10 days. Immunocytochemistry were performed with anti-synapsin antibodies and Alexa Fluor 594-conjugated α-bungarotoxin (Molecular Probes).

## Results

### Highly Efficient Neural Induction from Human Embryonic Stem Cells

The procedure for sMN differentiation from hESCs is schematically summarized in Figure 1A. As a first stage, NSCs were induced from KhES-1 (K1) hESCs with Noggin as described in Materials and Methods. After 10 days, neuroectodermal marker Nestin-positive-cell colonies were observed with high frequency (Figure 1B). In contrast, Desmin+ mesodermal or Alfa-fetoprotein (AFP)+ endodermal cells were not detected in K1-derived cell colonies (Figure 1B) but some positive cells were observed in K2- and K3-derived colonies (Figure S2A and C). Neuroectodermal cell colonies were dissociated by Collagenase and replated on PLL/LM-coated culture dishes for 7 days (total 17 days after neural induction). Nestin+ neural rosettes (NRs) emerged within 7 days after replating (Figure 1C). Strong AFP expression was still observed in some K2 and K3 colonies at the P2 stage but very weak AFP expression was detected in only a few K1 colonies (Figure 1C, Figure S2B and D). None or very few of either Desmin+ or Oct4+ colonies were observed in the culture of the three hESC lines (Figure 1C, Figure S2B and D). NR colonies in K1 hESCs were more compact than in the other two lines (Figure 1C, Figure S2B and D). Nestin+ neuroectoderm cells were also induced from monkey ESCs by Noggin (Figure 1F and G). Furthermore, we checked that immunostaining of Nestin was specific to neural stem cells because Nestin was expressed in some non-neural cells such as satellite cells [34]. Nestin+ cells at P2 did not express other differentiation markers, such as Oct4, Desmin, and AFP, while another neuroepithelial marker, Sox1 was expressed in Nestin+ cells (Figure S1). These results suggest that the immunostaining of Nestin was specific to neuroectodermal cells.

Recently, Dorsomorphin (DSM), a chemical inhibitor of BMP signaling, was used instead of Noggin for inducing myocardial differentiation in mouse ESCs [35]. Hence, we examined whether DSM was effective in neural induction of hESCs. K1 hESCs were cultured in N2B27 with 200 nM DSM in the same manner as Noggin treatment. DSM-treated K1 hESCs showed similar results in neural induction as those observed in Noggin-treated cultures (Figure 1D and E). These data indicated that the BMP inhibitor, DSM, could replace Noggin as a neural inducer.

### Differentiation of hESC-derived NSCs into Spinal Motor Neurons

**Figure 2:**
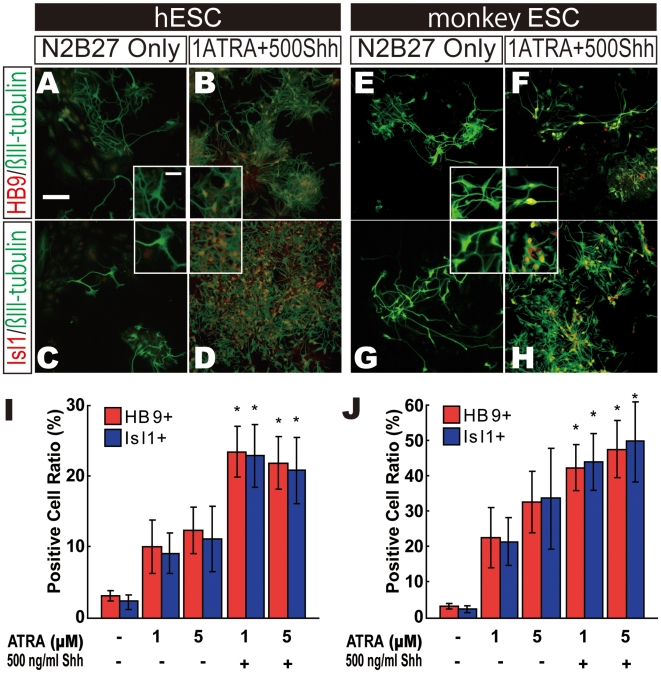
ATRA/Shh Promotes Differentiation to Spinal Cord Motor Neurons. Dissociated NRs, derived from hESCs or monkey ESCs, were cultured in the presence of ATRA and Shh for 7 days. When 1 µM ATRA and 500 ng/ml Shh were supplemented in the differentiation culture (B, D, F and H), larger numbers of βIII-tubulin+/HB9+ (B and F) and βIII-tubulin+/Isl1+ cells (D and H) were observed. Insets in A–H show high magnification. (I and J) The ratio of HB9- or Isl1-positive cells was calculated from the hESC differentiation culture (I) and monkey ESC differentiation culture (J). Scale bar in A indicates 100 µm. Inset scale bar indicates 10 µm. *p<0.05 (n = 5).

We thus chose the K1 hESC line for sMN differentiation because it showed the most effective neural induction by Noggin among the three hESC lines (Figure 1 and Figure S2). Dissociated NR colonies from either hESCs or monkey ESCs were plated on PLL/LM with Fibronectin (PLL/LM/FN)-coated dishes in N2B27 medium containing ATRA and Shh. Culture condition with 1 µM ATRA and 500 ng/ml Shh promoted cell proliferation more than did control culture (Figure 2A–H). Larger numbers of HB9+ or Isl1+ sMNs were observed in ATRA/Shh-treated culture at 7 days (Figure 2B, D, F, H, I and J). In contrast, very few HB9+ and Isl1+ cells were observed in control culture (Figure 2A, C, E, G, I and J). Higher concentration of ATRA (5 µM) had no effect on raising HB9+ or Isl1+ cell numbers in either hESC or monkey ESC-derived NSCs (Figure 2I and J).

**Figure 3:**
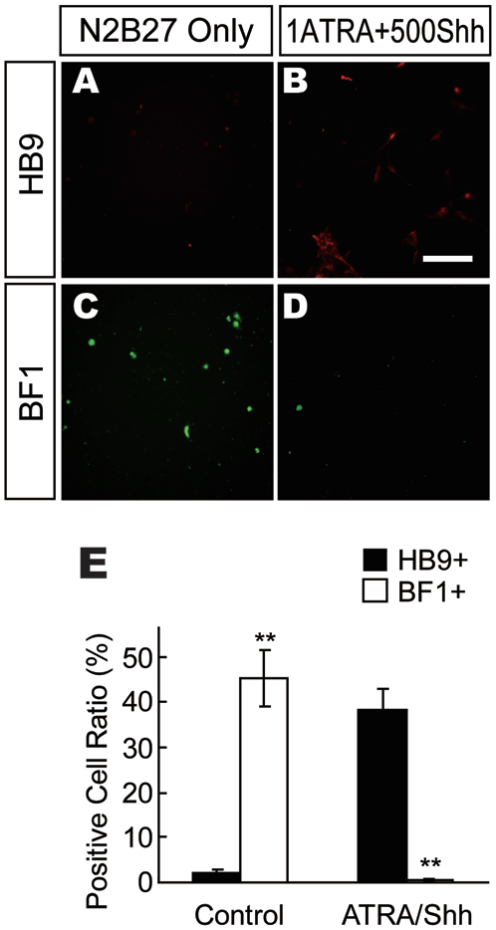
ATRA/Shh Strongly Changes Characteristics Rostrally to Caudally. (A–D) Spinal motor neuron marker HB9 and rostral neural marker BF1 in the control (A and C) or 1 µM ATRA and 500 ng/ml Shh culture conditions (B and D). (E) HB9 expression was strongly induced by ATRA/Shh treatment while BF1 expression was strongly repressed. *p<0.05 (n = 3).

We next examined whether regional characters were altered after ATRA/Shh treatment. BF1 is a rostral brain marker. Large numbers of BF1+ cells, but no HB9+ sMNs, were observed in control culture (Figure 3A, C and E). Conversely, many HB9+ cells, but no BF+ cells, were observed in ATRA/Shh-treated culture (Figure 3B, D and E). Similar results were observed in monkey ESC differentiation culture (Figure S3C). These findings suggested that neurons derived from NRs were characteristic of the rostral brain as a default state and that ATRA/Shh-treatment strongly changed the properties of neurons from the rostral brain to the caudal spinal cord.

**Figure 4:**
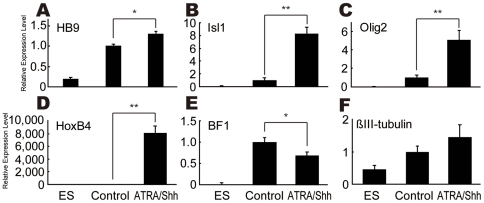
ATRA/Shh Induces the Expression of Spinal Cord-Specific and Motor Neuron-Specific Genes. (A–C) The expression of sMN-specific genes such as HB9, Isl1, and Olig2 was upregulated by the treatment of 1 µM ATRA and 500 ng/ml Shh. (D) A spinal cord-specific gene, HoxB4, was also upregulated in ATRA/SAG-treated cells compared to the control without ATRA/Shh. In contrast, the rostral brain marker BF1 expression was downregulated in ATRA/Shh-treated culture compared to the control culture (E). (F) There was no statistically significant difference in the expression levels of the neuronal marker βIII-tubulin between ATRA/Shh culture and the control. The gene expression levels in the control culture were defined as 1.0.*p<0.05, **p<0.005 (n = 3).

The gene expression levels of sMN markers such as HB9, Isl1 and Olig2 were upregulated 1.3-, 8.5- and 5.2-fold, respectively, by ATRA/Shh treatment (Figure 4A–C), and also a caudal brain marker, HoxB4, was drastically upregulated (Figure 4D). Similar results were obtained in monkey ESCs (Figure S4). On the other hand, BF1 expression level was downregulated by ATRA/Shh treatment (Figure 4E). These qtPCR results indicated that ATRA/Shh strongly induced sMN markers including spinal cord markers but repressed a rostral brain marker. No significant difference in the expression level of βIII-tubulin suggested that neurogenesis itself was not changed by ATRA/Shh (Figure 4F).

### The Small Molecule Drug, SAG, Produced More Spinal Neurons Than Did Shh

**Figure 5:**
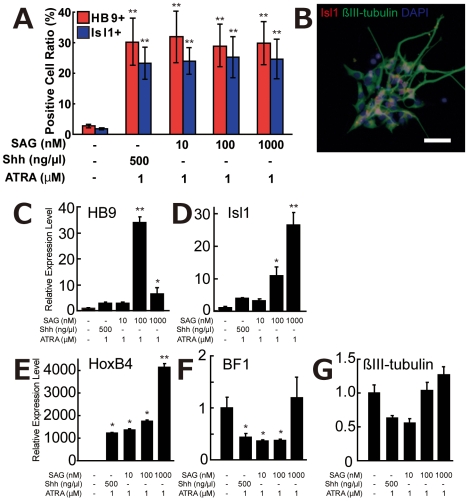
Shh Agonist Acts as a Motor Neuronal Differentiation Factor Equivalent to Shh Protein. (A) ATRA (1 µM) and SAG (10 to 1,000 nM) treatments as well as 1 µM ATRA and 500 ng/µl Shh treatment greatly increased both HB9+ and Is11+ cells. (B) Isl1/βIII-tubulin double-positive cells were observed in ATRA/SAG culture. White bar indicates 20 µm. (C–G) Quantitation of the gene expression levels in ATRA/SAG-treated culture. Two sMN-specific markers, HB9 and Isl1, were upregulated by ATRA/SAG treatment (C, D). A spinal cord marker, HoxB4, was also upregulated (E), while a forebrain marker, BF1, was downregulated by addition of ATRA/SAG (10 and 100 nM) (F). The change in expression of the neural cell marker βIII-tubulin was not statistically significant (G). *p<0.05, **p<0.005 (n = 4).

As described in the results of DSM, chemical reagents are very useful for constructing stable culture systems. In the same manner as DSM, we tried to use a Shh agonist, SAG, instead of recombinant Shh protein. SAG has been reported to directly inhibit the Smoothened receptor, which is constitutively inhibited by another Shh receptor, Patched, and activates downstream signals (Figure S3A) [36]. SAG (10 to 1,000 nM) was added into dissociated NRs culture with 1 µM ATRA to evaluate sMN differentiation-inducing activity. Ratios of HB9+ or Isl1+ cells were not significantly different between SAG-treated and Shh-treated cultures (Figure 5A). Representative staining using antibodies against Isl1 and βIII-tubulin is shown in Figure 5B and Figure S3B. The gene expression levels of sMN-specific genes (HB9 and Isl1) in ATRA/SAG culture were similar to or much higher than those in ATRA/Shh culture (Figure 5C and D). Rostral-caudal genes (HoxB4 and BF1) in ATRA/SAG culture were expressed in a manner similar to those in ATRA/Shh culture (Figure 5E and F). The highest concentration of SAG (1000 nM) did not suppress BF1 expression even in the presence of ATRA (Figure 5F), possibly because the effect of SAG changed in a dose-dependent manner [37]. The expression level of βIII-tubulin was not significantly changed by SAG (Figure 5G), suggesting that SAG did not affect neurogenesis. These results strongly indicated that the SAG molecule could be an alternative to Shh in sMN differentiation. Similar results were obtained in monkey ESCs (Figure S3C) and also in ENStem-A, H9-derived neural progenitor cells (Figure S5). These results suggest that our protocol for sMN differentiation could be applied to ESCs from other species and even to hESC-derived NSCs induced by different methods, although there were slightly different responses among a few hESC lines as described above.

### Mature Motor Neurons Are Electrophysiologically Functional and Form Synapses with Myotubes

**Figure 6:**
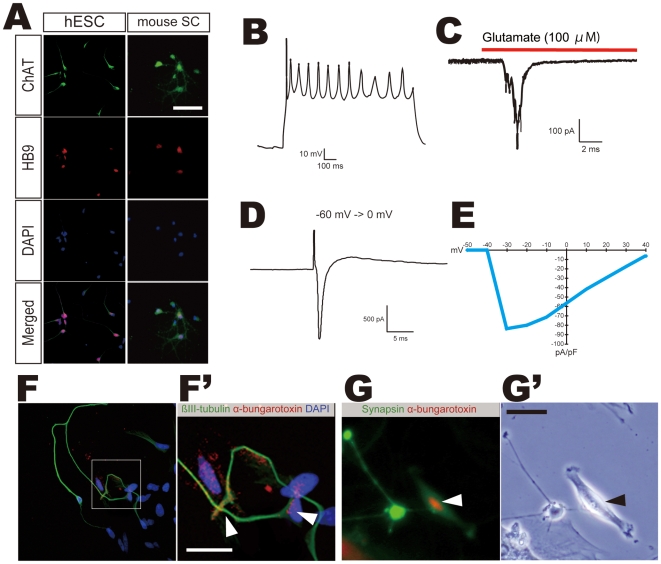
Mature Motor Neurons are Electrophysiologically Functional and Form Synapses with Myotubes. (A) A mature sMN marker, choline acetyl transferase (ChAT), was detected in hESC derived-neurons in the long-term culture. Mouse E14.5 spinal cord primary culture (mouse SC) was used as a positive control. White bar indicates 20 µm. (B) A current clamp recording showed repetitive firing of action potentials in response to the injection of a 1-second current pulse (50 pA). (C) A voltage clamp recording showed an inward current in response to the addition of 100 µM glutamate (V_holding_ = −60 mV). Red line indicates the glutamate application (D and E). A voltage clamp recording (D) and current density-voltage relationship (E) show inward currents in response to depolarizing voltage steps from −60 mV to 40 mV. (F and F') Terminals of βIII-tubulin+ neurons (green) were co-localized with the signals of an acetylcholine receptor marker α-bungarotoxin (red) on C2C12-derived myotubes (arrowheads). High magnification picture (F') indicates the area of open white square in F. (G and G') α-bungarotoxin signals (red, arrowheads) was detected nearby neural synaptic marker, Synapsin (green). Inset in G shows high magnification picture. Bars in F' and G' indicate 20 µm.

ATRA/SAG-treated hESC-derived sMNs were maintained in N2B27 medium supplemented with BDNF, NT-3, and GDNF for another 2 weeks or longer. During this culture term, hESC-derived neurons became sMNs expressing both HB9 and choline acetyl transferase (ChAT), which is the key enzyme required for the synthesis of acetylcholine (Figure 6A), suggesting that these double-positive cells were mature sMNs. Mature sMNs were further examined for these functions by electrophysiological analysis. Repetitive firing of action potentials in response to the injection of a 1-second current pulse (50 pA) was observed by a voltage clamp (Figure 6B). An inward current in response to the application of 100 µM glutamate was observed with a voltage clamp while holding voltage at −60 mV (Figure 6C). A voltage clamp recording and the current density-voltage relationship showed inward currents in response to depolarizing voltage steps from −60 mV to 40 mV (Figure 6C). These data indicated that mature functional sMNs could be produced from hESCs by our differentiation protocol.

Furthermore, we examined whether hESC-derived sMNs could form neuromuscular junctions. hESC-derived sMNs were co-cultured with mouse C2C12-derived myotubes for 4 to 10 days. Antibodies against either βIII-tubulin or Synapsin and fluorescence-conjugated α-bungarotoxin were used for detecting neuromuscular junctions. The signals of α-bungarotoxin were co-localized with axonal terminals of neurons (Figure 6F and arrowheads in Figure 6F') and also detected in myotubes adjacent to the signals of Synapsin, a neural synaptic marker (arrowheads in Figure 6G and G'). These data suggest that sMNs derived from hESCs had an ability to form neuromuscular junctions *in vitro*.

### Efficient sMN Production by Neurosphere Culture

**Figure 7:**
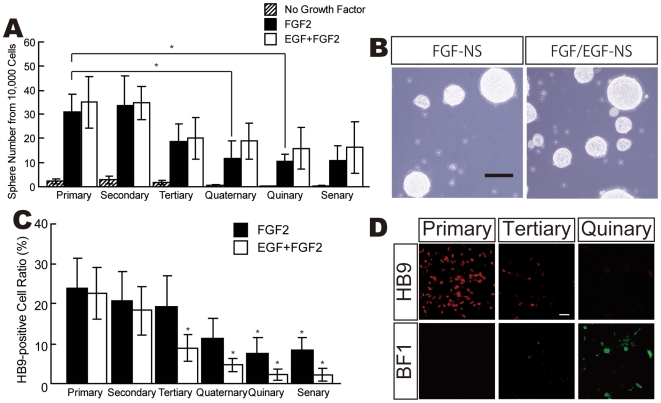
FGF2-treated Neurospheres Produce More sMNs Than Do EGF/FGF2-treated Neurospheres. (A) Both FGF2- and EGF/FGF2-treated neurospheres passaged though sphere numbers were gradually decreased depending on the number of passages. (B) Neurospheres in two different culture conditions showed no differences in morphology. Black bar indicates 100 µm. (C) The HB9+ sMN differentiation ratio in EGF/FGF2-treated neurospheres decreased more rapidly than in FGF2-treated neurospheres. (D) Immunocytochemistry of differentiated cells derived from EGF/FGF2-treated neurospheres. White bar indicates 20 µm. *p<0.05, **p<0.005 (n = 4).

Neurosphere culture is commonly applied to estimate multipotency of brain-derived NSCs as well as NSC propagation [15], [38]. In addition, neurosphere culture can be utilized for expansion of ESC-derived NSCs [33], [39]. Therefore, we next aimed to expand the number of our ESC-derived NSCs by neurosphere culture in growth factor-containing medium. Primary neurospheres derived from NRs were cultured in N2B27 medium containing 20 ng/ml FGF2 (NS-FGF) or both EGF and FGF2 (NS-EGF/FGF) at 100 cells/µl (Figure 7A). After 10 to 14 days, only a few neurospheres were observed in control N2B27 medium without growth factors (Figure 7A) and very few neurospheres in medium containing EGF alone (data not shown), whereas many neurospheres were formed in NS-FGF and NS-EGF/FGF and the formation rate showed no significant difference between NS-FGF and NS-EGF/FGF (Figure 7A). When SU5402, an FGF2 signal-specific inhibitor was added to neurosphere culture with FGF2-containing medium, neurosphere formation was completely suppressed (data not shown). Thus FGF2 was required for formation of NR-derived neurospheres. In addition, the formation rate of neurospheres decreased gradually according to the number of serial passages in both NS-FGF and NS-EGF/FGF (Figure 7A). There were no morphological differences between NS-FGF and NS-EGF/FGF (Figure 7B), but we found differences in the efficiency of sMN differentiation. Cells in both NS-FGF and NS-EGF/FGF could differentiate into HB9+ sMNs, though neurosphere-mediated HB9+ sMNs decreased gradually each passage (Figure 7C). However, HB9+ cells from NS-EGF/FGF drastically decreased after tertial passaging (Figure 7C and D) and rostral brain marker (BF1)-positive cells increased depending on the number of passages (Figure 7D). These results suggested that neurospheres in FGF2-containing media could maintain the ability to differentiate into HB9+ sMNs through ATRA/Shh treatment, but EGF in FGF2 condition could not. Using NS-FGF culture, sMN-producible neurospheres could be expanded. Simple calculation indicated that cells with a HB9+ differentiation potential of more than 20% expanded more than 30-fold by three passages of NS-FGF (Figure 7C). Similar results were obtained from monkey ES-derived neurospheres (Figure S6). Therefore, the NS-FGF culture method can stably expand a sMN population. Furthermore, frozen stocks of NS-FGF could maintain the ability to proliferate and differentiate into sMNs (data not shown).

### Purification of Spinal Motor Neurons by Gradient Centrifugation

Although our differentiation protocol resulted in highly efficient differentiation of sMNs, the differentiation efficiency was not 100% and non-sMNs co-existed in the culture. Therefore, we tried to isolate sMNs from the mixed population of differentiated cells derived from hESCs. At present, there are no identified cell surface markers for sMNs, but several reports have described purification of primary MNs from spinal cords of embryonic chick [20], [28], [30], rat [30], [40], [41], and mouse [29], [42], [43] using gradient centrifugation. To purify hESC-derived sMNs with naïve condition, discontinuous gradients were prepared by overlaying two different densities of Biocoll and then cells were put onto the gradient (Figure S7A). After centrifugation, two interfaces were carefully collected and immunochemistry was carried out. The results indicated that 78% of cells in interface 2 were HB9-positive (Table 1), indicating that the gradient centrifugation method enriched HB9+ sMNs more than 2.5-fold. Almost half of the cell population recovered in interface 2 (Table 1). In addition, monkey ESC-derived sMNs were also enriched by the same method, although results showed a lower recovery ratio (Figure S7B, Table S2). These data suggested that naïve sMNs could be obtained from mixtures of hESC/monkey ESC-derived differentiated cells without any cell surface markers or reporter transgenes.

**Table 1:** Enrichment of Naïve HB9+ Motor Neurons by Gradient Centrifugation.

	% of HB9+	% of Recovery
Total	29.5	(100)*
Interface 1	43.6	14
Interface 2	78.3	48
Pellet	25.6	38

Separated cells were immunostained with anti-HB9 and βIII-tubulin antibodies.

* Recovery ratio of total fraction shows 100%. The recovery ratio was calculated based on numbers of βIII-tubulin-positive cells. The ratio of HB9+ cells was calculated by the following formula: (number of HB9+ cells/number of total cells)×100.

## Discussion

Highly efficient sMN production from hESCs was achieved in four steps: 1) neural induction by Noggin or DSM, 2) neurosphere culture of hESC-derived NSCs, 3) sMN differentiation by ATRA/Shh or ATRA/SAG and 4) sMN purification by gradient centrifugation. Our protocol could be applied to not only KhES1 hESCs but also H9 hESCs. Although the methods of each step have been previously reported, we modified the procedures to improve the efficiency of differentiation and we showed that combinations of these techniques resulted in a large amount of naïve sMNs, which could be obtained especially by large-scale NSC-neurosphere culture and following isolation of the sMN population by gradient centrifuge. Enriched hESC-derived naïve sMNs are potentially available for not only tools in drug screening but also clinical applications for transplantation [44]. Our combination techniques using ESC-derived sMNs in this study might be applicable to other types of neural cells in the presence of proper extrinsic factors [9].

### Small Molecular Compounds in Differentiation Procedures

We showed that small chemical compounds such as DSM or SAG could replace recombinant proteins as factors necessary for neural induction or sMN differentiation (Figure 5 and Figure S3). The effect of DSM or SAG was equal to or greater than that of recombinant proteins. However, we found some differences between the immunostaining and the gene expression patterns (Figure 5A, C and D). Especially, the HB9 mRNA level decreased at 1,000 nM SAG/1 µM ATRA (Figure 5C), while HB9+ cell numbers did not change (Figure 5A). Also, the gene expression level of Isl1 was up-regulated in SAG dose-dependent manner (Figure 5D), although Isl1+ cells did not increased (Figure 5A). There are several reasons for these discrepancies. First, SAG could act as a Shh inhibitor at high concentration [37], [45]. Therefore, HB9 mRNA level might be down-regulated at high concentration of SAG (Figure 5C). Second, we speculate that 10 nM SAG is enough for the production of HB9 and Isl1-positive cells from almost all of Shh-responsive NSCs and high-dose of SAG (>100 nM) might up-regulate the gene expression of HB9 or Isl1 in sMNs (HB9- and Isl1-positive cells) by unknown effect of SAG. Although there are some unknown mechanisms of SAG, use of small molecules has several benefits: it can (1) reduce culture cost and (2) increase culture stability in comparison with the use of recombinant proteins. Therefore, small molecules are important for pharmaceutical applications such as drug screenings or safety tests in high-throughput systems.

### Neurosphere Culture for Expansion of ESC-Derived NSCs

In this paper, we showed that NS-FGF produced more HB9+ sMNs than did NS-EGF/FGF (Figure 7). The reasons why different effects occurred between two culture conditions are unclear. FGF2 was required for the formation of neurospheres using ESC-derived NSCs (Figure 7). Previous work has shown that FGF2 itself functions as a caudalizing factor [46]. Hence, the caudalization effect of FGF2 might help to maintain the potency of sMN differentiation of NS-FGF, whereas EGF might cancel the caudalization effect of FGF2 but not the neurosphere-forming effect, or simply a high dose of ATRA and Shh might be required for sMN differentiation from NS-EGF/FGF. BF1+ cells were detected from quinary NS-EGF/FGF (Figure 7D), but the exact reasons are uncertain. Therefore, further experiments are required for revealing reasons for this phenomenon in late passages of neurosphere culture.

The neurosphere technique we established could expand the numbers of NSCs more than 30-fold while preserving the potency of sMN differentiation (Figure 7 and Figure S5). Using this neurosphere culturing system, a large amount of either NSCs or sMNs could be prepared in appropriate quality. We are currently trying to determine culture conditions that increase or maintain HB9+ differentiation potency because it is necessary to maintain the potency of HB9+ sMN differentiation for large-scale experiments. In addition, these neurospheres were cryopreservable. Cryopreserved neurospheres might lead to much faster and more efficient sMN production.

### Enrichment of Naïve sMNs Derived from ESCs

It is important to purify or enrich particular cells from a mixed population from hESC-derived culture. Our differentiation method could produce sMNs with an average ratio of HB9+ cells of 30% in the total cell population; in other words, there were still other cell types such as NSCs, ventral interneurons and astrocytes in the cell culture. Certainly, it is possible to isolate a pure population using fluorescent reporters driven by a specific promoter or specific cell surface markers [7], [47]. However, no specific surface markers for sMNs have been reported. Thus, we tried enrichment of hESC-derived sMNs by gradient centrifugation. Our centrifugation-protocol raised the purity of sMNs from 30% to 80% at interface 2. Interface 1 contained a moderate ratio of HB9-positive cells, but the recovery rate was lower. This result suggests that more optimization is required for our protocol or that HB9-positive cells in these interfaces might differ from each other. In addition, the recovery ratio in monkey ESCs was much lower than in hESCs (Table 1 and Table S2), possibly because of a difference in sensitivity between dissociation enzymes of these species.

In conclusion, we have combined relatively simple methods and achieved an improvement in differentiation efficiency, amplification of hESC-derived NSCs and enrichment of hESC-derived naïve sMNs. Our techniques established here will be applicable for preparation of cells in large-scale culture systems, which are required for research toward drug discovery and development. We also propose that isolated hESC-derived sMNs could be important materials in both clinical and basic medical research.

Recently, ALS models using hESC-derived MNs have been reported [48]–[50]. When hESC-derived MNs were co-cultured with glial cells expressing mutant superoxide dismutase 1 (SOD1), the number of HB9-positive neurons decreased because of cell death by astroglial effect, namely, non-cell autonomous effect [48], [49]. On the other hand, another ALS model using mutant *SOD1*-transfected MNs showed that the HB9 promoter-driven mutant *SOD1* expression in hESC-derived MNs resulted in a decrease of MNs, suggesting cell-autonomous neuronal death [50]. Thus, the mechanisms of disease onset and progress are still controversial. Enriched population of hESC-derived MNs will be applicable for ALS modeling.

Functional sMNs generated by our protocol might be effective in applications such as personalized medicine by using cell banks of hESCs or human-induced pluripotent stem cells (hiPSCs). Patient-specific hiPSCs have been generated previously [51]. Among them, SMA patient's hiPSCs showed a cell-death phenotype [52]. Our sMN differentiation protocol will facilitate the generation of patient-specific hiPSCs for diagnosis of diseases and investigation of disease mechanisms, as well as drug screening. Moreover, extensive efforts to develop stem-cell based cell therapy have been carried out [14], [53]. Therefore, functional sMNs produced from hESCs or hiPSCs by our method will contribute to therapeutic application for MNDs.

## Supporting Information

Figure S1 Nestin immunostaining is specific to neural stem cells. (A–H, M–P) Nestin immunostaining never overlapped with other non-neural markers in Noggin-treated hESC cultures at 7 days of P2. (I–L) Another neural stem cell marker Sox1 immunostaining overlapped with Nestin immunostaining.
(8.54 MB TIF)

Figure S2 Other human embryonic stem cell lines were induced to neural cells by Noggin treatment. Two other hESC lines, KhES-2 or -3, were cultured with Noggin by the same method as that for KhES-1. Both hESC lines differentiated into Nestin-positive cells, although non-neural cells were observed at low frequency. White bar indicates 25 µm.
(7.34 MB TIF)

Figure S3 SAG acts the same as Shh on monkey ESC-derived neural stem cells. (A) SAG directly activates Smoothened, a Shh receptor, instead of inhibiting Patched suppression by Shh. Downstream transcription factors such as Gli are activated and serve to transcribe target genes which are originally transcribed by Shh signaling. (B) Isl1+ and βIII-tubulin+ sMNs were observed in monkey ES cell-derived neurons. White bar indicates 10 µm. (C) HB9+ and Isl1+ cells were detected in SAG-treated culture at the same ratio as Shh-treated culture. Rostral brain marker BF1-positive cells were also suppressed in SAG-treated culture as well as Shh-treated culture, while the control culture showed a high BF1+ ratio. *p<0.05, **p<0.005 (n = 4).
(1.90 MB TIF)

Figure S4 ATRA/Shh induces the gene expression of spinal motor neuron markers in monkey ESC-derived NSCs. The expression level of spinal cord marker HoxB4 (A), sMN markers such as Olig2, Isl1 and HB9 (B–D) were greatly upregulated by ATRA/Shh treatment. **p<0.005 (n = 3).
(1.19 MB TIF)

Figure S5 sMN differentiation from H9 hESC-derived NSCs by ATRA/SAG treatment. βIII-tubulin-positive cells were frequently observed in both the control and ATRA/SAG-treated ENStem-A culture for 14 days. Strong HB9-positive cells were observed in ATRA/SAG-treated culture while no HB9-positive cells were observed in the control culture. White bar indicates 25 µm.
(3.92 MB TIF)

Figure S6 Neurosphere culture of monkey ESC-derived neural stem cells. (A) The neurosphere-forming rate was gradually decreased during passaging in both FGF2 and FGF2+EGF conditions. (B) The HB9+ sMN differentiation ratio was gradually decreased during passaging in both FGF2 and FGF2+EGF conditions. However, the ratio decreased more rapidly in the FGF2+EGF condition than in the FGF2 condition. *p<0.05 (n = 5).
(1.21 MB TIF)

Figure S7 Monkey ESC-derived spinal motor neurons were purified by gradient centrifugation. (A) Discontinuous gradients were prepared by overlaying three different densities of Percoll-like reagents. ESC-derived sMNs were gently overlaid in the gradients. After centrifugation, two interfaces were carefully collected. (B) Cells were immunostained with both anti-HB9 and anti-βIII-tubulin antibodies on day 1 after separation. White bar indicates 20 µm.
(1.64 MB TIF)

Table S1 Primers used in this study. To eliminate the possibility of genomic DNA amplification, primers were designed with the Universal Probe Library Program (Roche, Germany, https://www.roche-applied-science.com/sis/rtpcr/upl/index.jsp). Before qtPCR, melt and standard curves of each primer set were generated to confirm that only a single amplicon was amplified with the same efficiency as the housekeeping gene β-Actin.
(0.07 MB DOC)

Table S2 Enrichment of naïve HB9-positive sMNs derived from monkey ESCs by gradient centrifugation. The same procedure and calculation as those for Table 1 were carried out.
(0.04 MB DOC)
