# Supplementary figures and images for: Highly Efficient Differentiation and Enrichment of Spinal Motor Neurons Derived from Human and Monkey Embryonic Stem Cells
Source: PLoS One. 2009 Aug 24;4(8):e6722. doi: 10.1371/journal.pone.0006722 (PMC2726947; doi:10.1371/journal.pone.0006722)

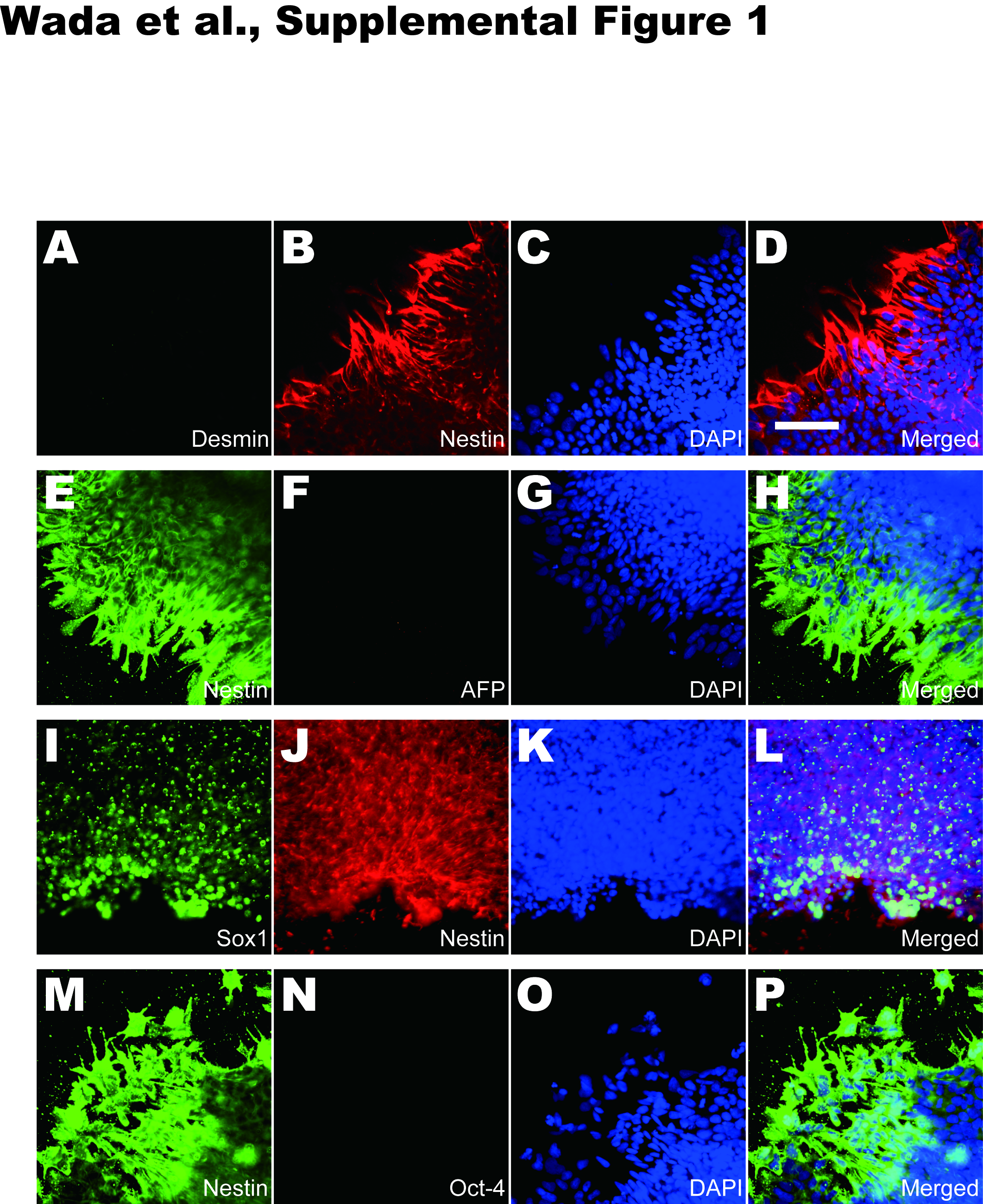

Supplement: Figure S1 — Nestin immunostaining is specific to neural stem cells. (A–H, M–P) Nestin immunostaining never overlapped with other non-neural markers in Noggin-treated hESC cultures at 7 days of P2. (I–L) Another neural stem cell marker Sox1 immunostaining overlapped with Nestin immunostaining. (8.54 MB TIF) [file pone.0006722.s001.tif]

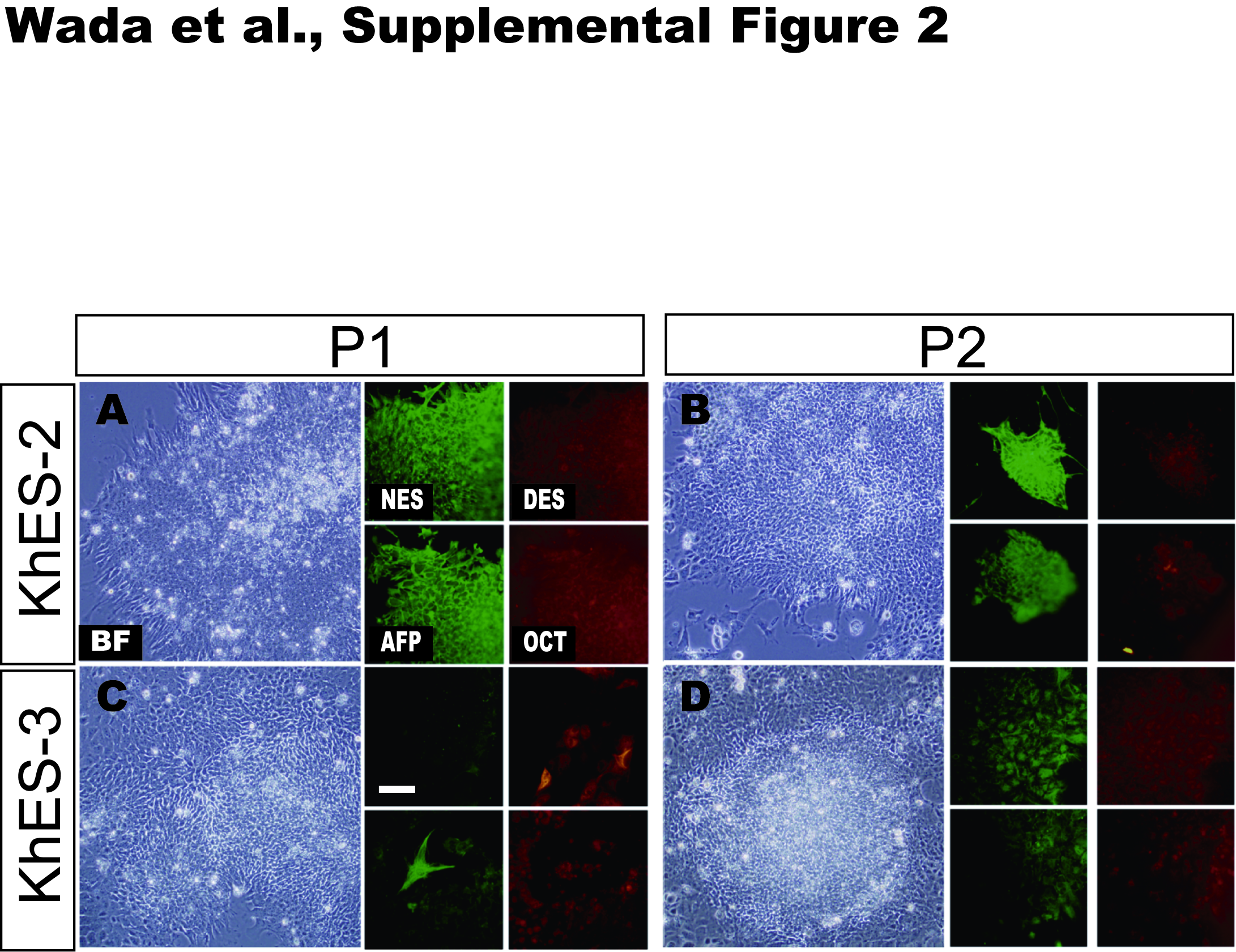

Supplement: Figure S2 — Other human embryonic stem cell lines were induced to neural cells by Noggin treatment. Two other hESC lines, KhES-2 or -3, were cultured with Noggin by the same method as that for KhES-1. Both hESC lines differentiated into Nestin-positive cells, although non-neural cells were observed at low frequency. White bar indicates 25 µm. (7.34 MB TIF) [file pone.0006722.s002.tif]

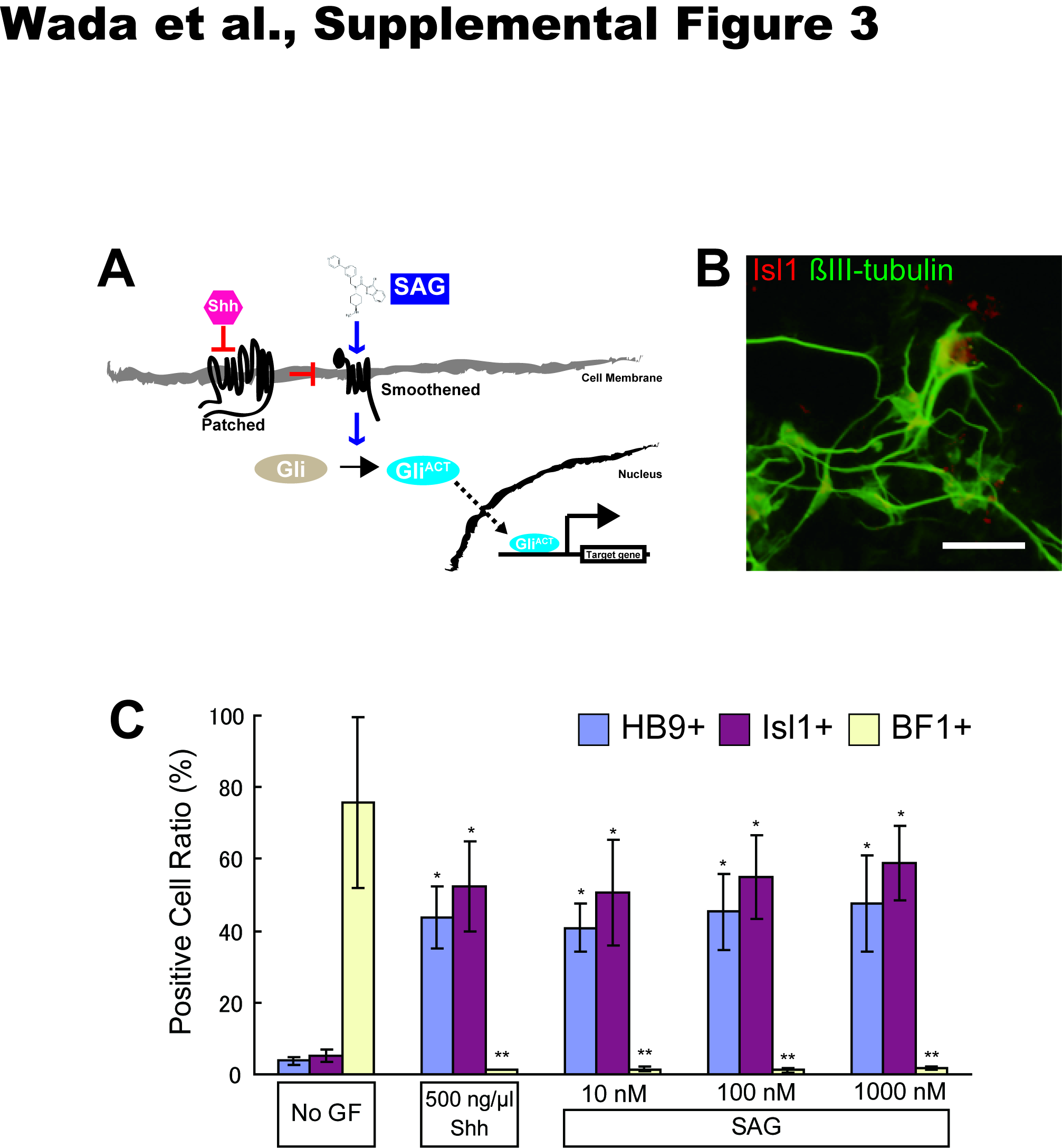

Supplement: Figure S3 — SAG acts the same as Shh on monkey ESC-derived neural stem cells. (A) SAG directly activates Smoothened, a Shh receptor, instead of inhibiting Patched suppression by Shh. Downstream transcription factors such as Gli are activated and serve to transcribe target genes which are originally transcribed by Shh signaling. (B) Isl1+ and βIII-tubulin+ sMNs were observed in monkey ES cell-derived neurons. White bar indicates 10 µm. (C) HB9+ and Isl1+ cells were detected in SAG-treated culture at the same ratio as Shh-treated culture. Rostral brain marker BF1-positive cells were also suppressed in SAG-treated culture as well as Shh-treated culture, while the control culture showed a high BF1+ ratio. *p<0.05, **p<0.005 (n = 4). (1.90 MB TIF) [file pone.0006722.s003.tif]

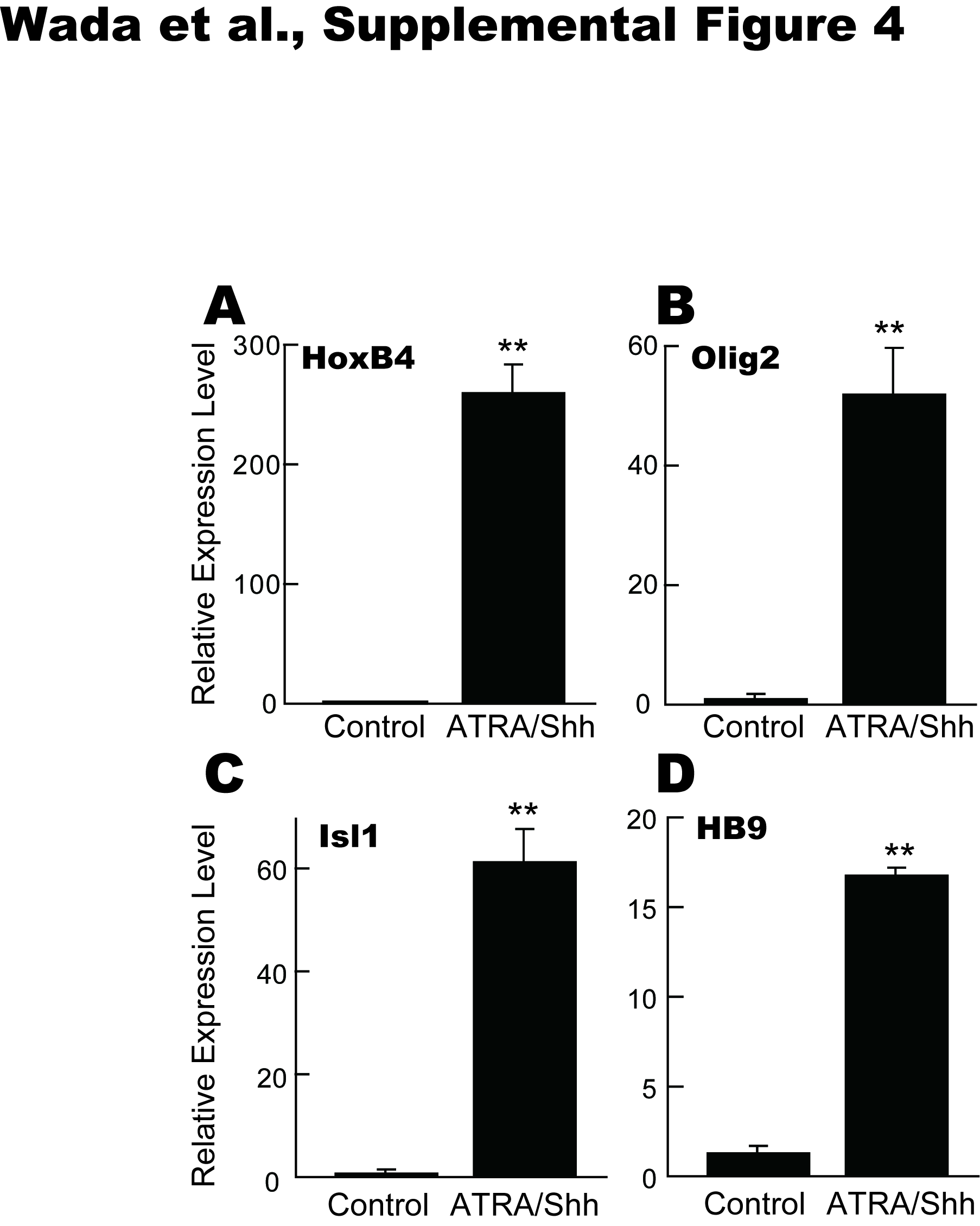

Supplement: Figure S4 — ATRA/Shh induces the gene expression of spinal motor neuron markers in monkey ESC-derived NSCs. The expression level of spinal cord marker HoxB4 (A), sMN markers such as Olig2, Isl1 and HB9 (B–D) were greatly upregulated by ATRA/Shh treatment. **p<0.005 (n = 3). (1.19 MB TIF) [file pone.0006722.s004.tif]

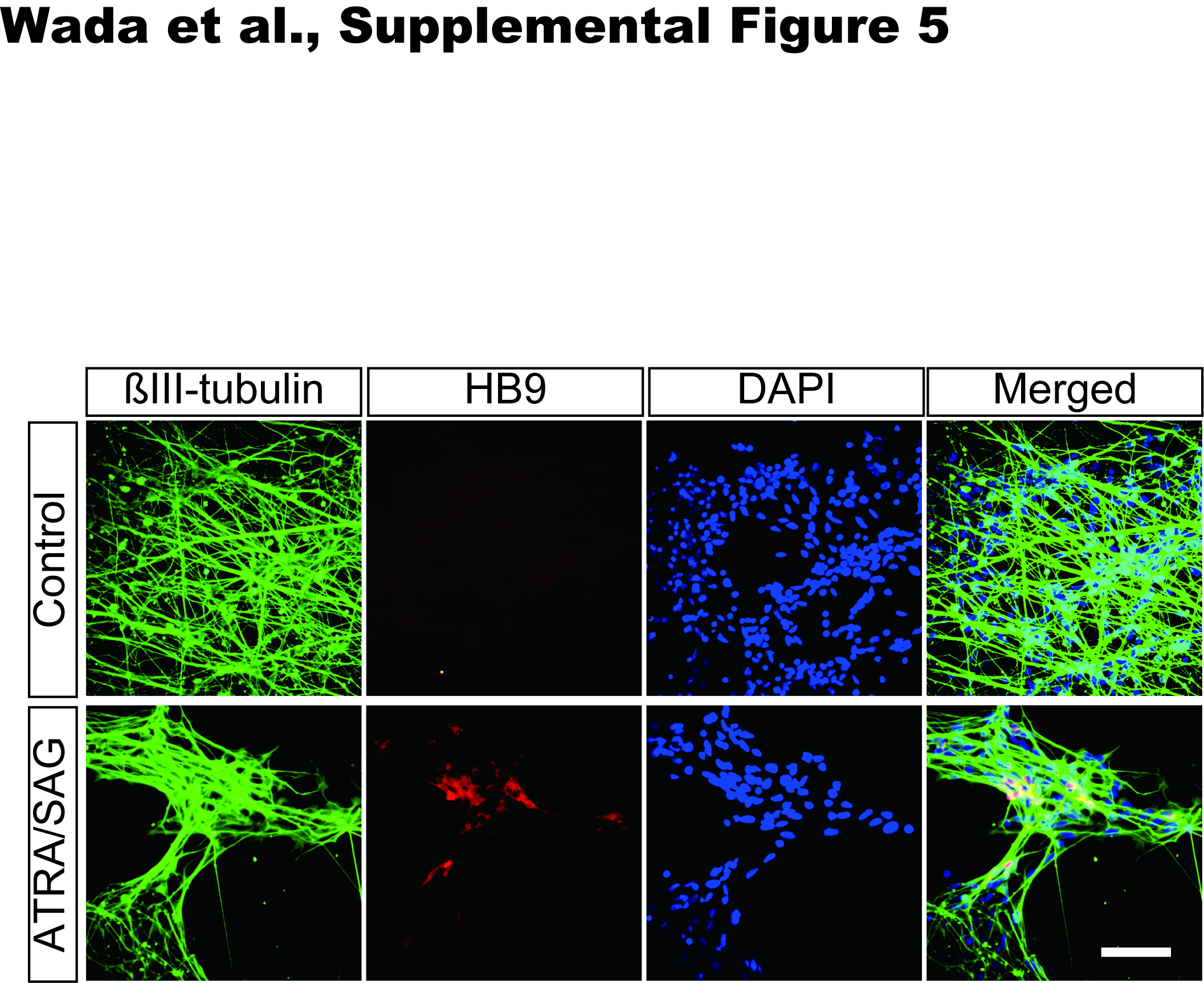

Supplement: Figure S5 — sMN differentiation from H9 hESC-derived NSCs by ATRA/SAG treatment. βIII-tubulin-positive cells were frequently observed in both the control and ATRA/SAG-treated ENStem-A culture for 14 days. Strong HB9-positive cells were observed in ATRA/SAG-treated culture while no HB9-positive cells were observed in the control culture. White bar indicates 25 µm. (3.92 MB TIF) [file pone.0006722.s005.tif]

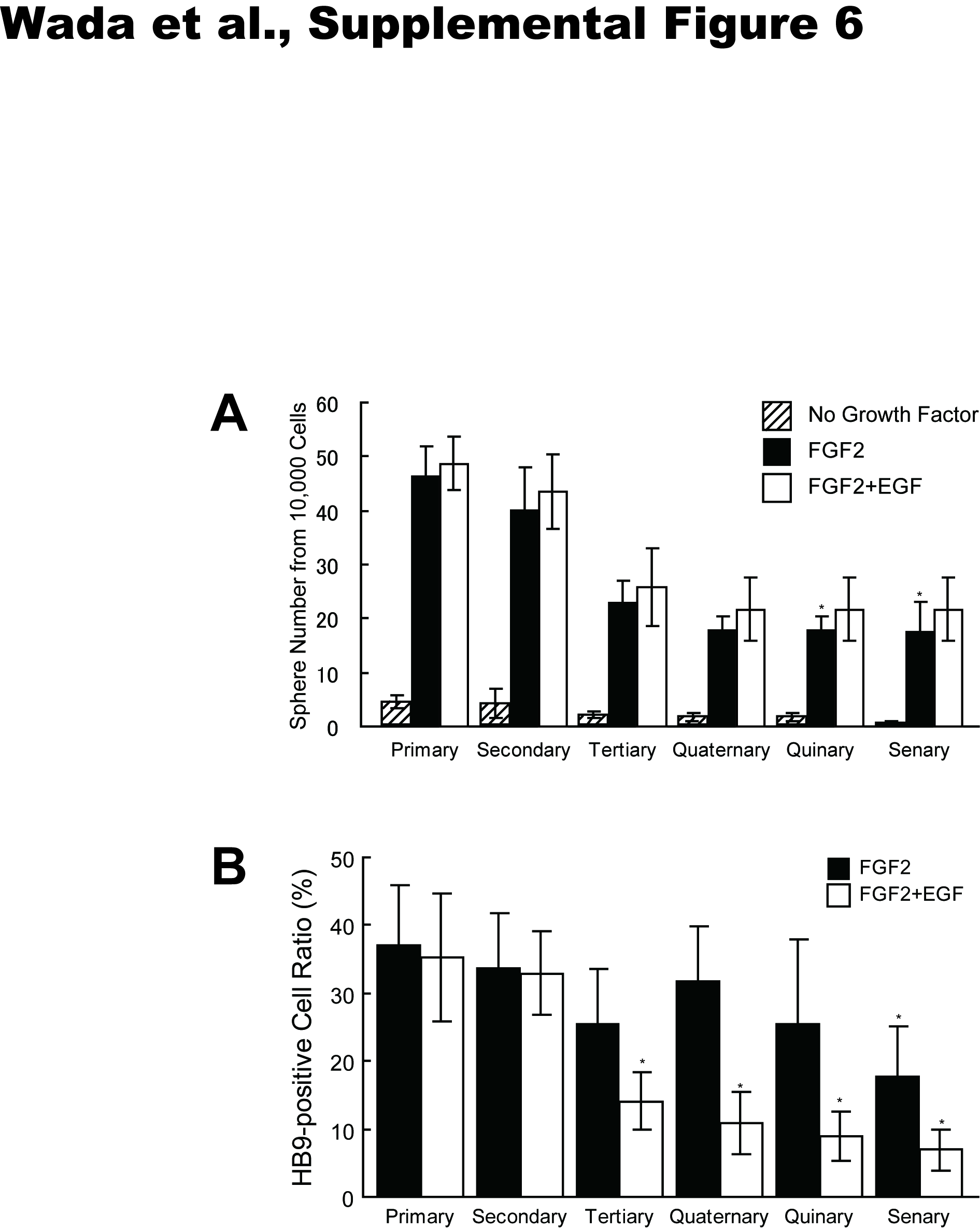

Supplement: Figure S6 — Neurosphere culture of monkey ESC-derived neural stem cells. (A) The neurosphere-forming rate was gradually decreased during passaging in both FGF2 and FGF2+EGF conditions. (B) The HB9+ sMN differentiation ratio was gradually decreased during passaging in both FGF2 and FGF2+EGF conditions. However, the ratio decreased more rapidly in the FGF2+EGF condition than in the FGF2 condition. *p<0.05 (n = 5). (1.21 MB TIF) [file pone.0006722.s006.tif]

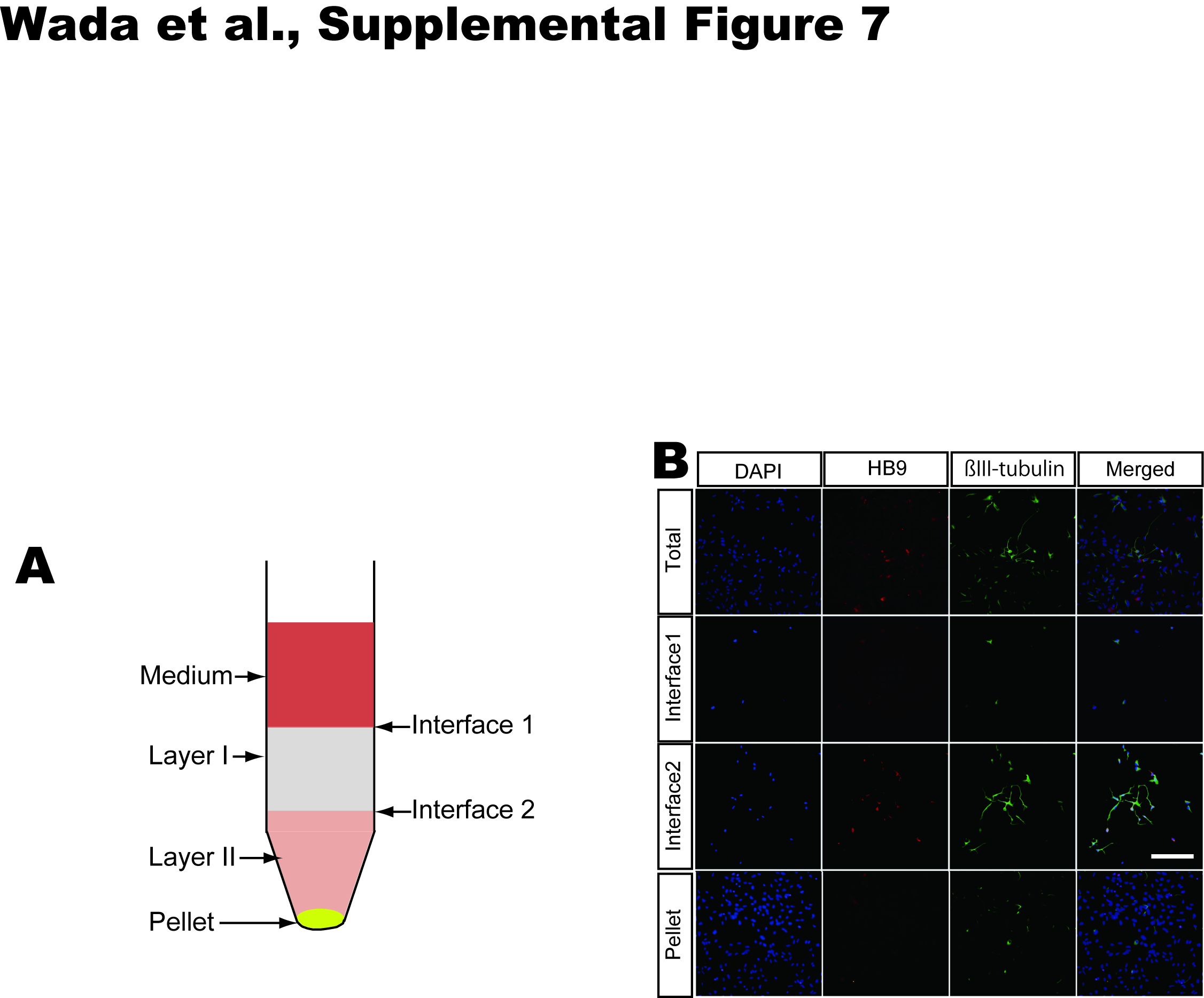

Supplement: Figure S7 — Monkey ESC-derived spinal motor neurons were purified by gradient centrifugation. (A) Discontinuous gradients were prepared by overlaying three different densities of Percoll-like reagents. ESC-derived sMNs were gently overlaid in the gradients. After centrifugation, two interfaces were carefully collected. (B) Cells were immunostained with both anti-HB9 and anti-βIII-tubulin antibodies on day 1 after separation. White bar indicates 20 µm. (1.64 MB TIF) [file pone.0006722.s007.tif]
