# Supplementary material for: Highly Efficient Differentiation and Enrichment of Spinal Motor Neurons Derived from Human and Monkey Embryonic Stem Cells
Source: PLoS One. 2009 Aug 24;4(8):e6722. doi: 10.1371/journal.pone.0006722 (PMC2726947; doi:10.1371/journal.pone.0006722)
Supplement: Table S1 — Primers used in this study. To eliminate the possibility of genomic DNA amplification, primers were designed with the Universal Probe Library Program (Roche, Germany, https://www.roche-applied-science.com/sis/rtpcr/upl/index.jsp). Before qtPCR, melt and standard curves of each primer set were generated to confirm that only a single amplicon was amplified with the same efficiency as the housekeeping gene β-Actin. (0.07 MB DOC) [file pone.0006722.s008.doc]

**Table S1. Primers used in this study**

| Marker | Gene | Orientation | %GC | Sequence | | Primer  Length (bp) | Amplicon  (bp) | Ex-Ex  Junction |
| --- | --- | --- | --- | --- | --- | --- | --- | --- |
| Motor Neuron | HB9 | FW | 53 | **TGCCTAAGATGCCCGACTT** | | 19 | 91 | Ex1-2 |
|  |  | RV | 53 | **AGCTGCTGGCTGGTGAAG** | | 18 |  |  |
|  | Islet1 | FW | 45 | **AAGGACAAGAAGCGAAGCAT** | | 20 | 85 | Ex4-5 |
|  |  | RV | 45 | **TTCCTGTCATCCCCTGGATA** | | 20 |  |  |
|  | Olig2 | FW | 53 | **AGCTCCTCAAATCGCATCC** | | 19 | 70 | Ex1-2 |
|  |  | RV | 53 | **ATAGTCGTCGCAGCTTTCG** | | 19 |  |  |
| Hindbrain  -Spinal cord | HoxB4 | FW | 53 | **CTGGATGCGCAAAGTTCAC** | | 19 | 138 | Ex1-2 |
|  |  | RV | 53 | **CGTGTCAGGTAGCGGTTGTA** | | 20 |  |  |
| Neuron | ßIII-Tubulin | FW | 61 | **GCAACTACGTGGGCGACT** | | 18 | 85 | Ex2-3 |
|  |  | RV | 55 | **CGAGGCACGTACTTGTGAGA** | | 20 |  |  |
| Rostral Brain | BF-1 | FW | 48 | **AGAAGAACGGCAAGTACGAGA** | | 21 | 169 | No |
|  |  | RV | 52 | **TGTTGAGGGACAGATTGTGGC** | | 21 |  |  |
|  | | | | | | | | |
| Internal Control | ß-Actin | FW | 50 | **ATTGGCAATGAGCGGTTC** | 18 | | 76 | Ex3-4 |
|  |  | RV | 50 | **GGATGCCACAGGACTCCAT** | 19 | |  |  |

To eliminate the possibility of genomic DNA amplification, primers were designed with the Universal Probe Library Program (Roche, Germany, <https://www.roche-applied-science.com/sis/rtpcr/upl/index.jsp>). Before qtPCR, melt and standard curves of each primer set were generated to confirm that only a single amplicon was amplified with the same efficiency as the housekeeping gene ß-Actin.
