# Supplementary material for: Highly Efficient Differentiation and Enrichment of Spinal Motor Neurons Derived from Human and Monkey Embryonic Stem Cells
Source: PLoS One. 2009 Aug 24;4(8):e6722. doi: 10.1371/journal.pone.0006722 (PMC2726947; doi:10.1371/journal.pone.0006722)
Supplement: Table S2 — Enrichment of naïve HB9-positive sMNs derived from monkey ESCs by gradient centrifugation. The same procedure and calculation as those for Table 1 were carried out. (0.04 MB DOC) [file pone.0006722.s009.doc]

**Table S2. Enrichment of naïve HB9-positive sMNs derived from monkey ESCs by gradient centrifugation**

|  | % of HB9+ | % of Recovery |
| --- | --- | --- |
| Total | 19.6 | (100) |
| Interface 1 | 32.5 | 1 |
| Interface 2 | 73.2 | 3 |
| Pellet | 1.8 | 91.3 |

The same procedure and calculation as those for Table 1 were carried out.
